# Can Biochemistry Usefully Guide the Search for Better Polymer Electrolytes?

**DOI:** 10.3390/membranes3030242

**Published:** 2013-09-17

**Authors:** J. Woods Halley

**Affiliations:** School of Physics Astronomy, University of Minnesota, 116 Church St SE, Minneapolis, MN 55455, USA; E-Mail: woods@woods1.spa.umn.edu; Tel.: +1-612-624-0395; Fax: +1-612-624-4578

**Keywords:** biochemistry, polymer electrolytes, lithium polymer batteries

## Abstract

I review some considerations that suggest that the biochemical products of evolution may provide hints concerning the way forward for the development of better electrolytes for lithium polymer batteries.

## 1. Introduction

Ion transport through the membranes of both polymer electrolyte membrane fuel cells and lithium polymer batteries has been limiting the development of both of these promising energy technologies for a decade or more. Empirical efforts to find more effective conducting membranes have had limited success. Theoretical and modeling efforts have clarified the mechanism of conduction in the case of lithium polymer membranes, but the insights obtained have not yet resulted in the development of membranes of the needed higher conductivities. This is partly because the mechanism depends on the very fundamental torsion forces in the hydrocarbon chains of the polymer, and these are not subject to modification simply by manipulation of the details of the hydrocarbon chemistry.

Through the 1980s and the 1990s, various models were proposed [1,2,3] to account for lithium conductivity in the polyethylene oxide (PEO)-based polymer electrolytes proposed for use in lithium polymer batteries, and though many works have reported simulation studies [4,5,6,7,8,9,10,11,12,13,14,15,16,17,18,19], consensus on the mechanism by which the lithium ions are transported has been slow to emerge for several reasons: Experimentally, it was shown quite early using nuclear magnetic resonance (NMR) studies that the lithium cations are moving through the amorphous fraction of the membrane, which is below its melting point and contains both amorphous and crystalline regions. Modeling the amorphous regions is a challenge in any case, but particularly because those regions are not in thermodynamic equilibrium. 

A more serious problem is that the molecular weights of the polymers in the membranes of engineering interest are large (as much as 1000 monomer units) in order to assure entanglement and dimensional stability. This has two consequences of relevance to the transport problem: It remains impractical to simulate a realistic sample of such long polymers using molecular dynamics techniques and, more fundamentally, this means that the reptation processes by which the centers of mass of such long polymers move are so slow that the motion of the centers of mass of the polymer chains is irrelevant to understanding the transport of the lithium. In practice, both fundamental scientific experiments and simulations have often been done on systems containing much shorter chains (10 monomer units is typical). For example, in one such recent study [20], the authors identified the relevant transport processes as “Diffusion of the cation along the polymer chain, cooperative motion of the cation with the polymer chain and cationic transfer between different polymer chains”. Under the engineering circumstances described, only the last of these is relevant to the problem of transport in a battery membrane. 

Nevertheless, some consensus has emerged concerning the nature of the processes by which lithium is transported. It is very similar to that described by the dynamic percolation models developed by Ratner and coworkers [2] in the 1980s: The lithium cations are captured by solvation shells consisting of ether oxygens of the polyethylene oxide chains. The energy wells in which the lithium is captured are usually much deeper than thermal energies (k*_B_*T) , so that the ion oscillates in its cage and moves slowly to and fro as the polymer chains undergo “crankshaft” motions. Very occasionally (on a scale of nanoseconds or more), the cage undergoes a fluctuation in its conformation, which permits a rapid (picosecond) transfer of the lithium ion to a cage on another chain, and a series of such events allows transport of the lithium across the membrane. The dependence of this picture on the crankshaft dynamics predicts the dependence of the lithium diffusion rate on the torsion forces in the backbone of the polymer. 

**Figure 1:**
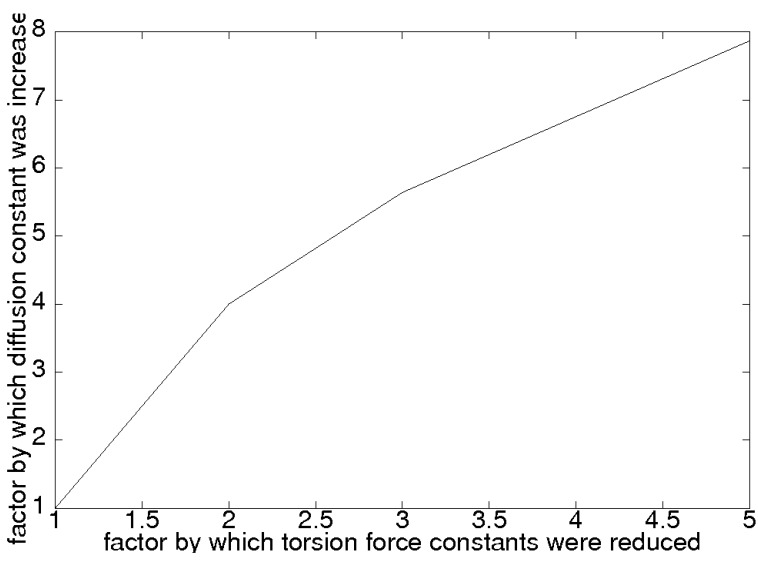
Simulated effect of reducing polymer torsion forces on the lithium diffusion constant. Reprinted with permission from [19]. Copyright 2005 AIP Publishing LLC.

About five years ago, we checked [19] this prediction within our molecular dynamics model for lithium transport in PEO by artificially reducing the torsion forces and recomputing the effective diffusion rate. The results are reproduced below in Figure 1 and show a clear dependence of the diffusion rate on the torsion force constant. While this seems to strongly indicate which parameter controls the transport in PEO electrolytes for lithium, it does not offer much hope to improve the transport properties by changing the details of the chemistry of the polymer; the torsion forces in the polymer backbone are of the same order of magnitude in many conceivable chemical variants of PEO. 

These considerations seem to suggest that, if the goal of 10^−^^3^ S/cm is ever to be approached for a lithium polymer battery electrolyte, then a quite radically different approach will be required. One such possibility, which has promising features, is the addition of room temperature ionic liquids to the traditional PEO-based electrolytes, as reported by Passerini and coworkers a few years ago [21,22,23,24]. It is unclear whether such mixtures will have the required dimensional stability, but this is an intriguing approach. Recently [25], the mechanical properties have been improved by adding wax particles in this approach. 

In this note, I briefly explore the possibility of another direction, based on the following qualitative observations: Evolution has produced extremely effective alkali ion channels, which are embedded in the lipid membranes of living cells and selectively pass potassium and sodium ions in and out of the cells. The channels are extremely complex, and it is unlikely that just reproducing them would lead to a useful material for a battery membrane. However, I note that (1) the embedding lipids are hydrophilic, which is a good feature for a lithium-metal battery electrolyte to have, and (2) the conduction mechanism is undoubtedly completely different from the one found in PEO-based electrolytes.

## 2. Protein PEO Mixtures

The only direct effort to use protein-based materials in a candidate electrolyte material that I have found is reference [26], which reports a PEO-soy protein isolate(SPI) film with lithium conductivities in the 10^−^^8^-10^−^^6 ^S/cm range, using lithium carbonate as the lithium salt. It is notable, however, that their SPI /PEO films had lithium ion conductivity that was higher than that of their PEO films, suggesting that the numbers might be increased by optimizing the choice of anion. The authors suggest that part of the increase in conductivity in the SPI/PEO films arises from the increase in the amorphous fraction in those films, relative to the PEO films, and, in part, from decreased ion pairing.

## 3. Membrane Proteins

Artificial alkali metal ion “hydrafile” channels have been synthesized , embedded in phospholipid membranes, and studied for their ion transport properties, as reviewed in [27]. The membranes are from 30 to 60 angstroms thick and have an effective resistance on the order of 10^9 ^ohms when biased to pass a sodium ion current. Extremely rough estimates of the conductivity are in the range of 10^−^^8 ^S/cm, which, of course, will not do at all if that is the end of the story. The films as measured are much thinner (nanometers) than electrolyte layers in existing lithium polymer batteries. If such thin films could be used, then the requirements on the conductivity would correspondingly be reduced by the ratio of the thicknesses. This is certainly problematical, but perhaps not totally impossible. Films like these have been engineered by nature to be extremely well sealed and hydrophobic. One should not assume that the films will behave in the presence of lithium cations in the same way that they do in the presence of sodium. There is evidence [28] that lipid bilayers crystallize in the presence of lithium carbonate, but not in the presence of sodium or potassium salts. The alteration of the bilayer structure in the presence of lithium salt is thought to be a possible mechanism for the effect of lithium salts in treating some forms of mental illness. A rigid bilayer lipid induced by lithium on the surface of a lithium metal layer and embedded with lithium ion conduction channels, such as the hydrafiles, described in reference [27], might be conceivable. Such a layer would have to comprise the solid electrolyte interface (SEI) layer, and more material might need to be added to provide a path for the conduction of the lithium cations to the cathode. A possible candidate for such a material might be a polymer containing more water than is tolerated in existing lithium polymer batteries. The water would increase the conductivity, and if the SEI were extremely hydrophobic, it might be kept away from the lithium anode.

## 4. Keeping out the Water

Introducing materials suggested by biochemistry into a lithium metal environment carries the risk of introducing water. In fact, the mechanism of transport for membrane proteins in cells is known to involve the passage of alkali ions in a one-dimensional arrangement interspersed with water molecules [30]. It appears not to be known whether similar mechanisms would permit alkali cation transport through membrane proteins in which water was replaced by small-molecule solvents other than water, such as those used in lithium air batteries. It has proven possible to produce protein-based materials that are free of water in the case of myoglobin [29], though I have found no reports of any similar achievement for membrane proteins. I note that PEO is water soluble and exists in aqueous solution in biochemical contexts; so, removing water (or at least loosely bound water) from protein materials that are candidates for electrolytes may also be possible.

## 5. Role for Simulation

Efforts to simulate the behavior of ion channels in biological membranes have an even longer history than that of similar efforts to simulate transport in battery electrolytes [20]. Unfortunately, they have not led to firm conclusions, either about the best simulation methods or about the mechanisms of transport [20]. However, with improved experimental understanding of transport mechanisms [30], better molecular dynamics force fields and much larger computers, it has become possible for simulation to play a constructive role in sorting out mechanisms and suggesting candidates in the search for a biochemically-derived polymer electrolyte for batteries. The positive role of simulation in the recently reported experimental elucidation of the transport mechanism in aquaporin [31], as well as the extensive molecular dynamics work reported by the White group [32,33,34,35] on membrane protein structure and function is consistent with this hope. Specifically, the search for membrane-like proteins or related polymers that bind to lipids, as they do in cells, but that function in nonaqueous solvents, can be significantly assisted by computational studies.

## 6. Conclusions

The main take home message that I hope to convey with these speculative remarks is that the stalled effort to find an improved electrolyte for a lithium metal battery might profit from a thorough exploration of the possibilities suggested by ion transport in biological systems. 
